# Can social isolation caused by physical distance in people with psychosis be overcome through a Phone Pal?

**DOI:** 10.1192/j.eurpsy.2020.53

**Published:** 2020-05-22

**Authors:** Mariana Pinto da Costa

**Affiliations:** 1 Unit for Social and Community Psychiatry (WHO Collaborating Center for Mental Health Services Development), Queen Mary University of London, London, United Kingdom; 2 Institute of Biomedical Sciences Abel Salazar, University of Porto, Porto, Portugal; 3 Hospital de Magalhães Lemos, Porto, Portugal

## Abstract

The current pandemic has forced many people into self-isolation and to practice social distancing. When people are physically isolated and distant from each other, technology may play a fundamental role by enabling social connection and reducing feelings of loneliness caused by this prolonged social isolation. In response to the COVID-19 pandemic, many mental health services worldwide have had to shift their routine face-to-face outpatient appointments to remote telepsychiatry encounters. The increased pressure on mental health services highlights the importance of community-led health-promotion interventions, which can contribute to preventing mental illness or their relapses, and to reduce the burden on health services. Patients with psychosis are particularly socially isolated, have sedentary lifestyles, and commonly face stigma and discrimination from the general population. At the same time, patients with psychosis value technology, are interested in, use and own smart-phones to digitally connect, and are satisfied with their use. Thus, among psychosocial interventions, a helpful resource may be “Phone Pal,” a complex intervention which facilitates remote communication between volunteers and socially isolated patients with psychosis through different smart-phone tools. While “Phone Pal” has been originally developed for people with psychosis, it may also be useful to the wider population, helping to overcome the social isolation caused by physical distancing, particularly in these times of widespread isolation. “Phone Pal” may be a potential public health resource for society, providing important support to those that may need it the most, and possibly benefit most from it.

Social isolation, sedentary lifestyle behaviors, and absence of close relationships with significant others are linked with worse outcomes, both in mental and physical health [1]. In light of this, attention should be paid to the effects of the current pandemic, which has forced much of the general population into self-isolation and to practice social distancing [2]. While the first requires staying at home, the latter restricts physical proximity to other people, and would probably be best termed as “physical distancing” [3].

When people are physically isolated and distant from each other, technology may play a fundamental role by enabling social connection and reducing feelings of loneliness caused by this prolonged social isolation. In particular, technology connects people, especially those who are hard to reach, and facilitates frequent and flexible communication [4]. During the current pandemic, technology may not only be a potential alternative to face-to-face connection, but may be the only way to respond to the extraordinary challenges, with many people at risk of total social isolation.

Despite this potential and the evidence supporting use of technology for remote communication, the long-standing debate on whether technology predominantly leads to social connection or social isolation remains. Concerns about the impact of technology have been prevalent for years, with worries about the negative effects when misused, and many questioning if people with mental illness are at all interested in using digital tools. Yet, the current necessity to make the most of technology as an ally may persuade these critics.

In response to the COVID-19 pandemic, many mental health services worldwide have had to shift their routine face-to-face outpatient appointments to remote telepsychiatry encounters, either through phone calls, video calls, or text messages [5]. This change has been necessary to guarantee the provision of the patients’ clinical care, while minimizing the risk of infection to both patients and clinical staff. Telepsychiatry is proving to be an effective resource while bridging psychiatrists, patients, and mental healthcare systems. Foremost, it enables all, especially those who must currently stay at home, to communicate with clinicians, be assessed, and receive therapy as required. The success of this mass transition to remote clinical consultations will be demonstrated if this approach is sustained beyond the aftermath of this pandemic.

The increased pressure on mental health services highlights the importance of community-led health-promotion interventions, which can contribute to preventing mental illness or their relapses, and to reducing the burden on health services. Encouragingly, despite these challenging times, people are keen to volunteer and give their free time to help others. While traditional volunteering is usually based on in-person contacts and on the active role played by the volunteer in supporting the patients to “do things” together (e.g., going out for a walk, having a coffee, doing shopping, etc.), this is not possible for many people. Therefore, remote volunteering via smart-phone may be a way to keep people socially connected and allow them to check in and chat with each other. Even when this role is more passive—for example, offering far away company—it can create a digital space that can sustain individuals in multiple ways. Although remote volunteering may require initial training and access to guidance and support from an organization, it may also broaden the recruitment of people that are interested and available to volunteer, and can promote real-world social relationships and positive attitudes towards people with mental illness. Smart-phones, already widespread across communities, present new opportunities to deliver interventions, monitor behavior changes, and increase communication between people who are physically distant from each other. While connected to the internet, smart-phones provide access to different communication methods (e.g., e-mails, WhatsApp, video calls, etc.), which are more or less synchronous.

The human nature of interpersonal communication is rooted in the face-to-face context of togetherness and synchronicity, where the sender and receiver are close by, possibly able to speak and hear each other and see facial expressions or gestures. In contrast, asynchronous communication removes the pressure to respond immediately, allowing the message to be retrieved in a different time and space by others. Nowadays, there is an increasing array of methods of interaction through technology that promote remote verbal (oral and written) and non-verbal communication. This allows various degrees of access to the other person in a more or less synchronous manner, and the concept of space is no longer restricted by a physical location.

A study exploring preferred technology-based communication tools (e-mail, phone, and face-to-face) in the general population reported that people communicate with their closest ties through different methods, and their choice largely depended on the availability of the tools, on having the skills to use them, and on whether these ties were local or not [6]. These findings highlight the role of accessibility, competence, and necessity when deciding which tool to use. Currently, technology is widely available and of upmost necessity; yet the demand for suitable training is rising and should not be neglected, especially to safeguard that those at a higher risk of social exclusion and marginalization do not become even more excluded due to a lack of digital competence. Training and support to those who need it should be a priority to avoid a digital divide with people missing out on opportunities to support one another.

People have long established contacts with persons they have not met before, as evidenced by the popular Pen Pal letters in the past. Technology may now enable and enhance how patients and volunteers establish and maintain a social relationship, despite being physically distant. This may positively impact the levels of social cohesion and community involvement, connecting people with mental illness to the wider society through the volunteers, and may help to change attitudes towards people with mental illness, thereby reducing the social distancing. Through smart-phone communication with a volunteer they do not know before, encouraging each other to have healthy lifestyles, may support patients to find new ways to interact, be reciprocal, and establish more secure attachments.

Patients with psychosis are particularly socially isolated, have sedentary lifestyle behaviors, and commonly face stigma and discrimination from the general population, who may keep them at a distance. Research has shown that patients with psychosis value technology, are interested in, use, and own smart-phones to digitally connect, and are satisfied with their use [7]. A systematic review on the use of smart-phones among patients with psychosis found no evidence of serious adverse effects of their use: no patients had an exacerbation of psychotic symptoms because of the smart-phone use. On the contrary, patients’ response was overall positive, and they found smart-phones to be easy to use and helpful in achieving their goals [8]. Another systematic review on the factors that affect the implementation of digital health interventions for people with psychosis reported that the complexity of the interventions, the lack of resources, and the costs hinder implementation, while accessibility and adaptability were facilitators [9].

Among psychosocial interventions, a helpful resource may be the “Phone Pal,” the protocol and study materials of which were reviewed favorably by the Research Ethics Committee of East of England—Cambridgeshire and Hertfordshire Research Ethics Committee (REC reference: 18/EE/0196); and is listed in the International Standard Registered Clinical/Social Study Number (ISRCTN17586238) clinical trial registry, which is recognized by the World Health Organization (WHO) and the International Committee of Medical Journal Editors (ICMJE).

The “Phone Pal” was developed for people with psychosis through a systematic process of consultation involving multiple methods and experts, including patients and community volunteers (the target population) who shared their views as per their needs throughout the development of the intervention. This complex intervention entailed remote communication through a smart-phone provided to both patients and volunteers, who were then able to use its different tools (texts, WhatsApp messages, e-mails, and audio and video calls) to communicate with each other.

The intervention was tested in a feasibility study with two phases. The first phase included London-based patients and volunteers and, after encouraging results (e.g., numerous expressions of interest to take part, including those outside London, and none of the participants dropping out), moved to the second phase where volunteers were recruited from across the United Kingdom. For up to 12 weeks, participants could conduct informal conversations and encourage each other to have healthy lifestyles. This feasibility study aimed to understand how the intervention, the training, and the access to support and supervision from a psychiatrist throughout work in practice, and to examine the potential impact of “Phone Pal” on people with psychosis and their volunteers, before deciding to proceed to a future larger trial to test effectiveness.

While “Phone Pal” has been originally developed for people with psychosis, it may be useful to the wider population, helping to overcome the social isolation caused by physical distancing, particularly in these times of widespread isolation. Volunteers can play an active role providing remote support to a neighbor or someone else distantly placed, such as a patient with a mental illness. A “Phone Pal” can be an invaluable public health resource for society, providing important support to those that may need it the most, and possibly benefit most from it. Prosocial digital behaviors that pull us together in a community model of empowerment are vital, and a “Phone Pal” could contribute to a society that works for everyone and where no one is left behind.

## References

[1] Sias PM, Bartoo H. Friendship, Social Support, and Health In: L’Abate L, editor. Low-cost approaches to promote physical and mental health: theory, research, and practice. New York, NY: Springer New York; 2007 p. 455–72.

[2] Fiorillo A, Gorwood P. The consequences of the COVID-19 pandemic on mental health and implications for clinical practice. European Psychiatry. 2020;63(1):e32. doi:10.1192/j.eurpsy.2020.35. 32234102 PMC7156565

[3] Wasserman D, Van der Gaag R, Wise J. The term ‘physical distancing’ is recommended rather than ‘social distancing’ during the COVID-19 pandemic for reducing feelings of rejection among people with mental health problems. European Psychiatry. 2020;63(1):e52 10.1192/j.eurpsy.2020.60. 32475365 PMC7287304

[4] Inkster B, Stillwell D, Kosinski M, Jones P. A decade into Facebook: where is psychiatry in the digital age? Lancet Psychiatry. 2016;3(11):1087–1090. 10.1016/S2215-0366(16)30041-4 27794373

[5] Pereira-Sanchez V, Adiukwu F, El Hayek S, Bytyçi DG, Gonzalez-Diaz JM, Kundadak GK, et al. COVID-19 effect on mental health: patients and workforce. The Lancet Psychiatry. 2020;7(6):e29–e30. 10.1016/S2215-0366(20)30153-X 32445691 PMC7239628

[6] Stern MJ. How locality, frequency of communication and internet usage affect modes of communication within core social networks. Inform Commun Soc. 2008;11(5):591–616. 10.1080/13691180802126778

[7] M Pinto da Costa, Chevalier A, Farreny A, Cassidy M, Leverton M, et al. How would patients with psychosis like to be in contact with a volunteer: Face-to-face or digitally? PLoS One. 2019;14(5):e0216929 10.1371/journal.pone.0216929 31095611 PMC6522036

[8] Firth J, Torous J. Smartphone apps for schizophrenia: a systematic review. JMIR Mhealth Uhealth. 2015;3(4):e102 10.2196/mhealth.4930 26546039 PMC4704940

[9] Aref-Adib G, McCloud T, Ross J, O’Hanlon P, Appleton V, Rowe S, et al. Factors affecting implementation of digital health interventions for people with psychosis or bipolar disorder, and their family and friends: a systematic review. Lancet Psychiatry. 2019;6(3):257–266. 10.1016/S2215-0366(18)30302-X 30522979

